# Cost for physician-diagnosed influenza and influenza-like illnesses on primary care level in Germany – results of a database analysis from May 2010 to April 2012

**DOI:** 10.1186/s12889-015-1885-0

**Published:** 2015-06-21

**Authors:** Birgit Ehlken, Anastassia Anastassopoulou, Johannes Hain, Claudia Schröder, Klaus Wahle

**Affiliations:** IMS Health, Erika-Mann-Str. 5, 80636 Munich, Germany; GlaxoSmithKline GmbH & Co. KG, Prinzregentenplatz 9, 81675 Munich, Germany; Present Address: Novo Nordisk Pharma GmbH, Brucknerstr. 1, 55127 Mainz, Germany; Department of General Medicine, University of Muenster, Domagkstr. 3, 48129 Muenster, Germany

**Keywords:** Seasonal influenza, Burden of disease, Cost, Complications, Risk factors

## Abstract

**Background:**

Seasonal influenza is one of the most significant infectious diseases in Germany; epidemic outbreaks occur every winter and cause substantial morbidity and mortality. However, published data from Germany on the current economic burden of influenza and the costs per episode are lacking.

**Methods:**

A retrospective database analysis was conducted using a longitudinal electronic medical records database (*IMS* Disease Analyzer). Patients with influenza, diagnosed by German office-based physicians using ICD-10 J09-11 (International Classification of Diseases, 10^th^ revision), who were observable in the database from 12 months before the index (diagnosis) date until 1 month afterwards, were included. The selection window, defined to cover two influenza seasons, was May 2010 to April 2012. Direct and indirect costs were evaluated from payer, patient and societal perspectives. Published unit costs and tariffs from Germany (2012) were used for the analysis.

**Results:**

A total of 21,039 influenza-attributable episodes in 17,836 adults, managed by primary care physicians (PCP) and 7,107 episodes in 6,288 children, managed by pediatricians, were eligible for analysis. The mean (±Standard Deviation (SD)) age of the adults with at least one episode was 46 (±18) years and 7 (±4) years in the children. The presence of clinical risk factors was documented for 39 % episodes in adults and 24 % episodes in children, with the most common being cardiovascular diseases in adults (29 %) and chronic respiratory diseases in children (23 %). Complications and severe symptoms accompanied the influenza-attributable episode (adults: 37 %, children: 54 %), bronchitis (adults: 16 %, children: 19 %) and acute upper respiratory infection (adults: 15 %, children: 21 %) being the most frequent. From a societal perspective, the total average mean cost (±SD) per episode was €514 (±609) in adults, where work days lost were the main cost driver (82 %), and €105 (±224) in children. Complications and severe symptoms increased the cost per episode versus episodes without by 1.7 times in adults (€684 (±713) vs. €413 (±510)) and nearly 3 times in children (€149 (±278) vs. €55 (±116)).

**Conclusions:**

Based on a large patient sample derived from representative PCP and pediatricians panels, our results demonstrate that seasonal influenza is associated with substantial clinical and economic burden in Germany.

**Electronic supplementary material:**

The online version of this article (doi:10.1186/s12889-015-1885-0) contains supplementary material, which is available to authorized users.

## Background

Influenza is an acute viral infection characterized by a range of symptoms, including sudden onset of high fever, cough, sore throat, headache, muscle and joint pain, severe malaise and a runny nose. Despite putting a high burden on patients and being of high socio-economic relevance to society, influenza is often regarded as an unproblematic and self-limiting disease, though, epidemic outbreaks, affecting all age groups, occur every winter and cause substantial morbidity and mortality. The World Health Organization (WHO) estimates that every year, 3–5 million severe cases and 250,000–500,000 deaths are caused by the influenza virus worldwide [1].

Influenza imposes a considerable economic burden as well. In Italy, the reported cost of seasonal influenza epidemics (1999–2008) ranged from €0.3 to €2.7 billion; the yearly average was €1.4 billion [2]. In Germany, the sole cost of the 1996 influenza epidemic was estimated at €2.6 billion (DM 5 billion) including direct costs, due to hospitalization, outpatient care and medication, as well as indirect costs, due to loss of work force productivity and absenteeism from work [3]. According to an analysis from the United States (US), the direct costs relating to hospitalization and treatment are twice as high when complications occur [4]. Groups at high risk of complications are the elderly, infants and patients with chronic illnesses [1]. Indirect costs arising during an influenza season were found to make up 80–90 % of the total costs in Germany and France [3, 5].

Vaccination has been shown to be the most cost-effective intervention in preventing infection and subsequently reducing the associated financial burden [6, 7]. Safe and effective influenza vaccines prevent 44–73 % of influenza symptoms among healthy adults [8] and reduce deaths by 80 % in the elderly [1]. WHO recommends annual vaccination (in order of priority) for nursing-home residents, elderly individuals, people with chronic medical conditions and other high-risk groups, such as pregnant women and children aged between 6 months to 5 years [1]. The European Union (EU) recommends that the national vaccination committees follow the definition of risk groups and “older age groups” as recommended by the European Centre for Disease Prevention and Control (ECDC) [9]. In contrast, the Centers for Disease Control and Prevention (CDC) in US recommends influenza vaccination to the general population over 6 months [10]. Currently, the German Standing Committee on Vaccination (STIKO) recommends annual influenza vaccination for high-risk individuals, particularly those above 60 years of age and pregnant women, in order to lower the disease burden associated with seasonal influenza [11].

Healthy working adults traditionally have not been included in the recommendations for influenza vaccination. In 1996, the economic burden induced by productivity losses as the result of seasonal influenza in Germany was estimated to be €2.3 billion (DM 4.4 billion) [3]. However, these values might now be higher and published data on the current economic burden of influenza and the costs per influenza episode in Germany are lacking.

The current retrospective database analysis aims to fill the research gap, which has existed in Germany for over 10 years, by describing and quantifying the economic burden associated with seasonal influenza in children and adults at the primary care level.

## Methods

### Study design

This was a retrospective cost-of-illness analysis, using data from a longitudinal electronic medical records (EMR) database (*IMS* Disease Analyzer (DA)) in Germany to estimate the cost per influenza or influenza-like illness (ILI) episode. Direct cost for physician visits including referrals to specialists, drugs and hospitalizations were considered from payer perspective and co-payments as well as transportation expenses from patient’s perspective. Indirect cost due to work days lost was taken from societal perspective into account. Subsequently, the economic burden of influenza was extrapolated for the whole of Germany by applying the derived cost per episode to independently published estimates of influenza-associated excess physician consultations per season thereby considering influenza seasons of the last 10 years.

### Database

The *IMS* DA comprises longitudinal patient-level data from the physician-practice data systems (EMR) of office-based physicians. The *IMS* DA Germany captures data from a representative sample of 3,002 unique physicians, 2,357 unique practices and includes 15.5 million patient records. The current retrospective database analysis was based on the data from office-based primary care physicians (PCP panel) and pediatricians (pediatricians panel) of *IMS* DA Germany and covered 1,409 PCPs (*i.e.* 2.3 % of the 60,800 PCPs) and 221 pediatricians (*i.e.* 3.6 % of the 6,100 office-based pediatricians) across Germany. Comparisons with external data sources (*e.g.* data from Statutory Health Insurance (SHI)) have underlined the validity and representativeness of using the German *IMS* DA in pharmacoepidemiological and pharmacoeconomic studies [12].

### Population and episode

The population of this cost study consisted of patients with influenza or ILI episodes, diagnosed using ICD-10 J09-J11 (International Classification of Diseases, 10^th^ revision) during the selection window from May 2010 to April 2012. This population allowed the description of influenza and ILI episodes per month over the 24-month observation period from an epidemiological viewpoint.

In order to evaluate risk factors, complications and severe symptoms as well as resource use associated with an influenza or ILI episode, the following additional inclusion criterion was applied: patients were continuously observable in the database for at least 12 months before and at least 1 month after the diagnosis (index) date of the episode. Notably, the 12-month observation period to diagnosis (pre-index period) was used to define patient’s risk status.

An influenza or ILI episode was defined as the day of physician visit when the index diagnosis was made (index date), together with a related period of 14 days before and 14 days after that date. In order to distinguish between two subsequent episodes in a single patient, a period of at least 29 days between index dates was required. Complications and severe symptoms (according to the ICD-10 codes, please refer to Tables 1 and 2) were associated to an influenza or ILI episode in the case they were reported at index date or within 14 days before or after the index date.

**Table 1:** Demographic and clinical characteristics^a^ - patients with influenza or ILI episodes between May 2010 and April 2012 in Germany

	Adults	Children
	N episodes = 21,039	N episodes = 7,107
Gender female % (n)	55.3 (11,629)	47.2 (3,353)
Age in years		
Mean ± SD	45.8 ± 17.8	6.9 ± 3.6
Min - Max	17.0–101.8	1.1–16.0
Age groups % (n)		
<5 years	0.0 (0)	33.7 (2,394)
5–16 years	0.0 (0)	66.3 (4,713)
17–59 years	79.2 (16,666)	0.0 (0)
≥60 years	20.8 (4,373)	0.0 (0)
Insurance status at index date % (n)		
Statutory Health Insurance	93.9 (19,765)	92.2 (6,550)
Private insurance	6.1 (1,274)	7.8 (557)
Relevant pre-period risk factors^b,c^ (as defined by STIKO)		
Weakened immune system		
Cancer treatment (C00-C97)	1.9 (392)	0.1 (9)
HIV/AIDS (B20-B24)	0.1 (24)	0 (0)
Chronic illness		
Any chronic disorders of respiratory organs (J45, J46, J44, J38, J35)	9.5 (1,995)	22.9 (1,629)
Any cardiovascular disorders (I10-15, I20-25, I26-I28, I30-I52, Z99.0-Z99.1)	28.5 (6,003)	0.8 (55)
Any cerebrovascular disorders (I60-I69, G45)	1.9 (397)	0.1 (5)
Diabetes and other metabolic diseases (E10-E14)	8.9 (1,876)	0.1 (7)
Hepatic disorders (B15-B19, K70, K75)	0.5 (114)	0.1 (6)
Renal disorders (N17- N19, Z99.2)	0.1 (196)	<0.1 (1)
Any neurological disorders (G04-G05, G20-G21,G30, G35, G40-G41)	1.4 (292)	0.8 (57)
Proportion of episodes with at least one of the above mentioned pre-period risk factors plus age ≥60 years in the adult patient group % (n)	38.8 (8,165)	24.3 (1,727)

^a^Analysis at episode level; ^b^Pre-period: 15–365 days before the index-date of the episode; ^c^Relevant diagnoses were documented as complications/severe symptoms of influenza if they occurred within 14 days before or after the index date. Equivalent diagnoses which could be both risk factors and complications were unified within the “risk factor” term and documented as such if they were made within 365 days before the index date.

*HIV/AIDS* Human immunodeficiency virus/acquired immune deficiency syndrome, *ILI* Influenza-like illness, *SD* Standard Deviation, *STIKO* German Standing Committee on Vaccination

**Table 2:** Occurrence (% (n)) and type of complications and symptoms (by ICD-10 code)^a,b^ - patients with influenza episodes between May 2010 and April 2012 in Germany

	Adults	Children
	N episodes = 21,039	N episodes = 7,107
Bronchitis (J20-J22, J40-J44)	16.0 (3,368)	18.9 (1,346)
Acute upper respiratory (J06)	14.6 (3,063)	20.8 (1,479)
Sinusitis (J01, J32, J31)	6.0 (1,271)	8.9 (636)
Tonsillitis (J03)	2.4 (498)	7.8 (556)
Laryngitis (J04-J05)	2.3 (491)	4.2 (295)
Pneumonia (J12-J18)	1.6 (336)	5.4 (381)
Ear infection (otitis) (H65-H67)	0.9 (190)	13.4 (955)
Bronchiectasis (J47)	0 (0)	0 (0)
Cardiovascular disorders (I30, I32, I33, I40, I42, I20-I24)	0.2 (52)	0 (0)
Cerebrovascular disorders (I60-I66)	0.1 (22)	0 (0)
Neurological disorders (G00-G05)	<0.1 (6)	0.1 (5)

^a^Analysis at episode level; ^b^Relevant diagnoses were documented as complications/severe symptoms of influenza if they occurred within 14 days before or after the index date. Equivalent diagnoses which could be both risk factors and complications were unified within the “risk factor” term and documented as such if they were made within 365 days before the index date.

*ICD* International Classification of Diseases

Information on influenza vaccination was not analysed because documentation in the EMR data is ambiguous. In practice, the influenza vaccine is often removed from stock without specific prescription and the documented diagnosis ICD-10 Z25.2, although indicating a need for influenza vaccination, does not clearly ensure that the vaccination was administered.

### Analyses

Analyses were conducted at the episode level and therefore included patients with one or more influenza episodes. Data from the PCP and pediatrician panels (patients’ age ≥17 years and 0–16 years, respectively) were evaluated separately.

#### Number of influenza episodes

The number of influenza or ILI episodes was analysed per month over the complete 24-month observation period.

#### Demographic and clinical characteristics

Socio-demographic characteristics were presented by patient’s age, gender and insurance status. The clinical risk factors were described during the 365 days before the index date of the episode and were defined according to STIKO. The ICD-10 codes for underlying diseases are listed in Table 1. Patients were considered “at risk” if they had at least one of the diagnoses mentioned in Table 1 or were ≥60 years.

Relevant complications were identified according to published literature [13, 14]. In addition, as the occurrence of influenza-specific symptoms (for example, bronchitis and pharyngitis) has an impact on the course of the illness and associated costs, these symptoms were also considered in case they were recorded during the influenza and ILI episode (defined as diagnosis ICD-10 J09-J11, index date ±14 days). Complications and severe symptoms of an influenza infection were not explicitly discriminated in this cost analysis. From a health economic perspective, the differentiation between complications and severe symptoms is not relevant for the calculation of episode-related cost. Therefore, complications and severe symptoms are summarized in the “Discussion” section under the term “complication”. The relevant diagnoses are listed in Table 2.

Relevant diagnoses were documented as complications/severe symptoms of influenza if they occurred within 14 days before or after the index date. Diagnoses which could be both risk factors and complications were unified within the “risk factor” term and documented as such if they were made within 365 days before the index date.

#### Resource and cost analysis

Resource consumption, due to the symptoms and complications during influenza or ILI episodes (index date ±14 days), was derived from data on physician visits, referrals to other specialists (cardiologists, pneumologists, neurologists, otorhinolaryngologists, laboratory and radiology, and others), drug prescriptions, hospital referrals and sick notes available in the data source. Only resource use and sick notes connected with influenza (ICD-10 J09-J11) or complications and symptoms of influenza were considered for the analysis. As no information was available on sick notes for caregivers or the use of over-the-counter (OTC) drugs in the database, these parameters were not considered in the analysis.

Direct costs from the payer perspective include cost of physician visits, referrals to other specialists, hospital admissions with relevant diagnoses and cost of drugs for treatment of influenza or its complications and symptoms. Direct costs from patient perspective included patient’s expenses for transportation and co-payment for drugs. The cost of additional physician visits per episode, which were not covered by the budget-per-quarter reimbursed by the payers, was considered as direct cost from the societal perspective.

The analysis of direct costs was based on resource utilization data, expressed as the number of services used. Cost was reported as the number of consumed services combined with the unit cost of each resource. The number of resources consumed was multiplied by the specific unit cost for each service. Published unit costs and tariffs from 2012 were used as follows: official medical fee schedule (Einheitlicher Bewertungsmaßstab (EBM)) 2012 for office-based physician visits; the German Diagnosis-Related Groups (G-DRG) Catalogue 2012 plus base rate in 2012 (€2,992) provided by the National Association of Statutory Health Insurance Funds for inpatient stays; the German Rote Liste 2012 for drugs [15–18] (see Additional file 1 for details). For the calculation of drug cost, the price per prescribed package was considered. Analysis of drug co-payments was performed for patients aged 18 years and above. Patient and family expenses for transportation were calculated based on the number of physician visits multiplied by patient expenses as described in the published literature [19].

For the calculation of indirect costs, the number of days absent from work was multiplied with the average productivity loss per day. The *IMS* DA provides information on the number of days recommended to be absent from work recorded on the sickness notes. The monetary value of productivity loss per day for employed persons was calculated based on gross wage data and the number of persons in dependent employment (data source: German Federal Statistical Office 2012 [20]) in accordance with German recommendations on health economic evaluation 2008 [21].

The time horizon for cost analysis per episode was 29 days (index date ±14 days). The complete influenza-related resource use and sick leave starting during the episode were considered for cost analysis. No censoring at day 14 after the index date was undertaken. The results of the cost analysis were stratified according to age and the presence of risk factors or complications and severe symptoms. Discounting of costs was not applied.

### Estimation of the annual economic burden of physician-attended influenza

The annual economic burden was estimated by multiplying cost per episode, derived from the current analysis, with the number of influenza-associated excess consultations per influenza season published by The Robert Koch Institute (RKI) [22]. The RKI has developed an approach to describe the seasonal variation of medically attended acute respiratory infections and the estimation of their excess, attributable to influenza, based on virological data from sentinel practices around Germany. A detailed description of the model was published by an der Heiden *et al.* in 2013 [23].

The minimum and maximum numbers of excess consultations for children and adults used for the extrapolation was based on the figures available for the last 10-year influenza seasons (2005–2014) in Germany.

### Statistics

All analyses were conducted using descriptive statistical methods. Total number, number of missing values, minimum, median, maximum, mean, standard deviation (SD) and the lower and upper limit of 95 % confidence intervals (CI) for the mean were provided for continuous variables. For categorical parameters, the absolute and relative frequencies were reported. The available information was analyzed “as reported” and missing values were not replaced.

Data were analyzed using *SAS* version 9.2.

## Results

Between May 2010 and April 2012, 42,822 episodes in 37,471 patients ≥17 years were identified in the PCP panel and 12,295 episodes in 11,049 patients <17 years were identified in the pediatricians panel. The vast majority of episodes (PCP panel: 96.4 %, pediatricians panel: 92.7 %) was classified as ICD-10 J11 “influenza, virus not identified”. The remaining episodes were classified as ICD-10 J9 “Influenza due to certain identified influenza virus” (PCP panel: 0.8 %, pediatricians panel: 4.0 %) or ICD-10 J10 “Influenza due to other identified influenza virus” (PCP panel: 2.8 %, pediatricians panel: 3.3 %). For the majority of patients, the diagnosis was made based on clinical symptoms and was not confirmed by laboratory tests.

**Fig. 1:**
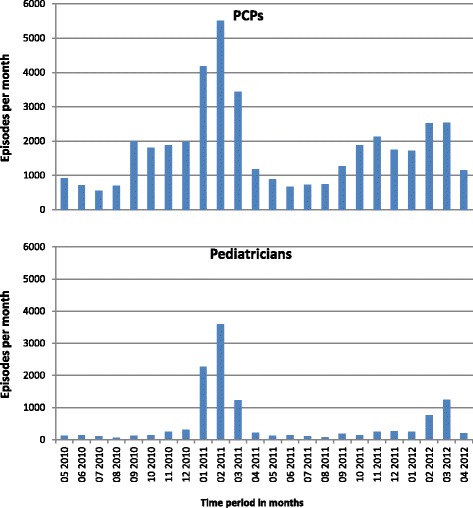
Number of influenza/ILI episodes per month reported in the primary care physicians (PCP) and the pediatricians panels between May 2010 and April 2012 in Germany

The peak of influenza episodes for the season 2010/2011 was observed in February 2011 and in March 2012 for the season 2011/2012 (Fig. 1).

### Population for analysis

A total of 21,039 influenza episodes in 17,836 adults managed by PCPs and 7,107 episodes in 6,288 children managed by pediatricians were eligible for the analysis of clinical characteristics, resource use and cost according to the inclusion criterion.

The vast majority of patients had one influenza episode (adults: 87.2 %, children: 90.0 %) or two influenza episodes (adults: 9.9 %, children: 7.9 %) within the 24-month observation period.

### Demographic and clinical characteristics

The demographic and clinical characteristics are summarized in Table 1.

The mean (±SD) age of patients was 45.8 (±17.8) years in the PCP panel and 6.9 (±3.6) years in the pediatricians panel. The majority of patients, 93.9 % and 92.2 % respectively, was insured by SHI.

The presence of clinical risk factors was documented for 38.8 % episodes in adults (including age ≥60 years) and 24.3 % episodes in children. The most common clinical risk factors were cardiovascular diseases in adults (28.5 %) and respiratory diseases in children (22.9 %).

### Complications and symptoms

Complications and severe symptoms frequently accompanying influenza episodes are presented in Table 2. Complications and severe symptoms accompanied the influenza-attributable episode in 37.4 % of adults and in 53.7 % of children. Bronchitis (adults: 16.0 %, children: 18.9 %) and acute upper respiratory infections (adults: 14.6 %, children: 20.8 %) were the most common symptoms. In adults, sinusitis (6.0 %) and in children, ear infections (13.4 %) and sinusitis (8.9 %) were the most frequent complications. Pneumonia was recorded in 1.6 % of adults and 5.4 % of children. At least one of these conditions was reported during the majority of episodes in patients without risk factors (adults: 55.8 %; children: 70.9 %).

### Resource use and cost

The mean (±SD) number of physician visits was 1.2 (±0.5) per episode in adults and 1.5 (±0.8) per episode in children. Referrals to other specialists were recorded during 13.6 % episodes in adults and 7.4 % episodes in children. The percentage of patients hospitalized because of influenza or its complications was similar in the PCP and pediatricians panels, 0.7 and 0.9 % respectively. Drug prescriptions relating to either the influenza diagnosis or complications/severe symptoms were issued during 48.1 % episodes in adults and 55.1 % episodes in children (Table 3). The mean (±SD) number of drug classes prescribed was 0.8 (±1.0) per episode in adults and 1.0 (±1.1) per episode in children. In adults, the most frequently prescribed drug class was expectorants (25.7 % episodes) followed by analgesics (20.1 % episodes) and macrolides (15.9 % episodes). In children, the most frequently prescribed drug class was analgesics (46.7 % episodes) followed by expectorants (33.0 % episodes) and nasal decongestants (32.1 % episodes).

**Table 3:** Drug prescriptions related to influenza and complications (%(n))^a^ - patients with influenza episodes between May 2010 and April 2012 in Germany

Drug class	EphMRA ATC code	Adults	Children
		N episodes = 21,039	N episodes = 7,107
Drug prescription no		51.9 (10,914)	44.9 (3,190)
Drug prescription yes		48.1 (10,125)	55.1 (3,917)
Prescribed drugs			
Analgesics	N02B1	9.9 (999)	0.3 (12)
	N02B2	10.2 (1,031)	46.4 (1,817)
Antirheumatics non-steroid, plain	M01A1	6.9 (699)	0.9 (36)
Nasal decongestants	R01A7	3.8 (380)	32.1 (1,256)
Antitussives, plain	R05D1	12.4 (1,251)	9.0 (352)
Cold preparations	R05A0	7.9 (804)	0.6 (24)
Expectorants	R05C0	25.7 (2,599)	33.0 (1,292)
Tetracyclines & combinations	J01A0	5.0 (506)	0.2 (9)
Broad spectrum pencillin oral	J01C1	6.5 (659)	5.9 (230)
Cephalosporins oral	J01D1	5.5 (560)	9.9 (389)
Macrolides & similar type	J01F0	15.9 (1,612)	6.7 (262)
Oral fluoroquinolones	J01G1	6.7 (681)	0
Influenza antivirals	J05B4	5.7 (575)	6.6 (260)

^a^Analysis at episode level

*ATC code* Anatomical Therapeutic Chemical code, *EphMRA* European Pharmaceutical Marketing Research Association

Sickness certificates were issued during 60.7 % episodes in adults (12,779 of 21,039 episodes) and in <0.1 % episodes in children (3 of 7,107 episodes). Adults missed up to 6 days from work during 46.7 % episodes; 7 or more days were missed during 14.0 % episodes. The overall mean (±SD) number of working days lost was 3.3 (±4.4) days in adults.

The mean (±SD) total cost per episode, from a societal perspective, was €514 (±609) in adults and €105 (±224) in children (Table 4). The total cost per individual episode exceeded €1,000 in about 12.6 % episodes in adults. The main cost driver in adults is indirect cost (82 %) followed by direct cost from payer perspective.

**Table 4:** Total cost per influenza/ILI episode (€)^a^ - patients with episodes between May 2010 and April 2012 in Germany

	Adults		Children	
	N episodes = 21,039		N episodes = 7,107	
	Mean (SD)	Median (Min – Max)	Mean (SD)	Median (Min – Max)
Physician visits	32 (2)	31 (31–36)	35 (5)	31 (31–42)
Referrals to other physicians	2 (9)	0 (0–129)	1 (5)	0.0 (0–88)
Hospital admissions with relevant diagnoses	14 (178)	0 (0–6,002)	17 (193)	0 (0–3,411)
Drug treatment	11 (51)	0 (0–2,216)	13 (33)	<1 (0–708)
**Subtotal - direct cost payer**	59 (188)	35 (31–6,060)	66 (201)	42 (31–3,527)
Co-payments drugs	7 (12)	0 (0–245)	0 (0)	0 (0–0)
Transportation	8 (4)	6 (6–66)	9 (5)	6 (6–54)
**Subtotal - direct cost patient**	15 (15)	12 (6–275)	9 (5)	6 (6–54)
Physician visits	16 (24)	0 (0–393)	30 (35)	31 (0–292)
**Subtotal - direct cost not covered by payer**	16 (24)	0 (0–393)	30 (35)	31 (0–292)
Work days lost	424 (566)	385 (0–11,664)	1 (62)	0 (0–4,230)
**Subtotal - indirect cost**	424 (566)	385 (0–11,664)	1 (62)	0 (0–4,230)
**Total cost from societal perspective**	**514 (609)**	**421 (37–11,878)**	**105 (224)**	**72 (37–4,534)**

^a^Analysis at episode level

*SD* Standard Deviation

**Fig. 2:**
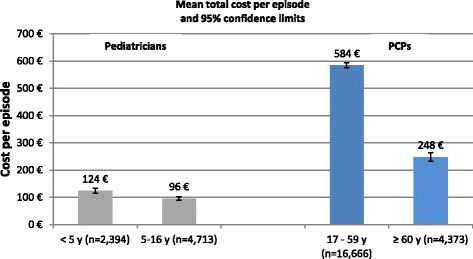
Total cost per influenza/ILI episode (€) stratified by age group in patients with episodes between May 2010 and April 2012 in Germany (PCP: primary care physicians)

Stratified by age group, the mean (±SD) total cost in adults was higher in patients between 17 and 59 years (€584 (±608)) and was considerably lower in patients ≥60 years (€248 (±535)). In children, the total costs were more comparable in all age groups, ranging from €96 (±214) in children aged 5–16 years to €124 (±242) in children younger than 5 years of age (Fig. 2).

**Fig. 3:**
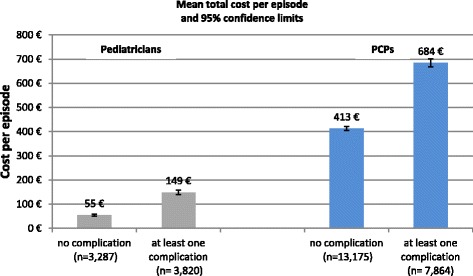
Total cost per influenza/ILI episode (€) stratified by complications (yes/no) - patients with episodes between May 2010 and April 2012 in Germany (PCP: primary care physicians)

Complications and severe symptoms increased the mean (±SD) total cost per episode 1.7 times in adults (€684 (±713) with complications or severe symptoms vs. €413 (±510) without) and nearly 3 times in children (€149 (±278) vs. €55 (±116)) (Fig. 3).

### Annual economic burden of physician-attended influenza

Based on the last 10 annual influenza seasons in Germany, excess consultations per season ranged from 0.2 to 2.2 million in children and from 0.3 to 5.6 million in adults. Multiplying the number of influenza-associated excess consultations by total cost per episode of €105 (for children) and €514 (for adults) results in an annual economic burden of physician-attended seasonal influenza of €0.2– €3.1 billion from societal perspective (Table 5).

**Table 5:** Estimated cost per influenza season [22]

	Estimated number of excess consultations per age group and season		Cost per age group and season in Billion €		Estimated total cost per season in Billion €
Season	Age group	Age group	Age group	Age group	All age groups
	0–14 years	15+ years	0–14 years^a^	15+ years^b^	
2013/2014	220,000	550,000	0.023	0.283	0.306
2012/2013	2,150,000	5,560,000	0.226	2.858	3.084
2011/2012	530,000	1,600,000	0.056	0.822	0.878
2010/2011	810,000	1,320,000	0.085	0.678	0.763
2009/2010	1,070,000	1,870,000	0.112	0.961	1.073
2008/2009	1,335,000	2,925,000	0.140	1.503	1.643
2007/2008	500,000	705,000	0.053	0.362	0.415
2006/2007	1,285,000	1,435,000	0.135	0.738	0.873
2005/2006	550,000	310,000	0.058	0.159	0.217
2004/2005	2,210,000	3,240,000	0.232	1.665	1.897

^a^number of excess consultations multiplied by € 105

^b^number of excess consultations multiplied by € 514

## Discussion

The current retrospective database analysis describes and quantifies the burden of seasonal influenza in adults and children at the primary care level in Germany. The aim of the manuscript was to estimate the annual economic burden of influenza in Germany. Due to the lack of up to date cost data, we have assessed the resource consumption related to the treatment of episodes classified as influenza or ILI (ICD-10 J9-J11) by primary care physicians. The aim was not to evaluate the incidence of confirmed influenza in Germany. Due to this reason, we have used figures published by RKI to extrapolate the cost per episode to the economic burden in Germany. Our analysis, based on a very large sample of patients with clinically diagnosed influenza, showed that the annual economic burden of seasonal influenza can reach €3.1 billion. Interestingly, a substantial burden occurs in adults without risk factors for complications and thus outside the recommended groups for seasonal influenza vaccination.

Our analysis of patients diagnosed with influenza between May 2010 and April 2012 in Germany reveals the monthly distribution of influenza/ILI episodes and accurately describes the 2010/2011 and 2011/2012 influenza seasons [24, 25]. In the season 2010/2011, the peak of influenza episodes was observed between January and March. In the following season (2011/2012), the peak occurred between February and March. However, this was much flatter compared to the 2010/2011 season. Our observations of monthly influenza/ILI episode distribution correspond to the patterns in reportable positive influenza diagnoses and practice consultations recorded by the RKI acute respiratory disease (ARE) monitoring system.

Influenza episodes are frequently accompanied by complications. We observed complications in 37.4 % of adults and in 53.4 % of children. Three circumstances, however, make it challenging to compare our findings with the published literature. Firstly, results often refer only to hospitalized patients; secondly, the types of reported complications differ; and thirdly, the time period patients are followed-up for varies widely. In a retrospective US managed care database analysis, complications were documented in 65 % of adults within a 12-month follow-up period [4], as compared to our findings in adults (37.4 %). The difference can be explained by the large variation in length of follow-up (12 months in the US analysis vs. 1 month in the present analysis) and the range of investigated complications, which was broader in the US analysis and included musculoskeletal diseases, diabetes and infectious diseases in addition to respiratory disorders. In the present analysis, the most frequent complications in children managed by pediatricians were respiratory tract infections (20.8 %) and otitis media (13.4 %); this pattern is comparable with published literature [26, 27]. In a review of pediatric influenza managed in the community and primary care in Western Europe, frequent complications included otitis media (between 0 and 41 %), bronchitis (between 5 and 10 %), pharyngitis (between 31 and 58 %) and the common cold (between 15 and 20 %) [26].

Some conditions can put patients at higher risk for developing complications. Overall, we found the most frequent risk factors for complications to be cardiovascular diseases in adults (29 %) and respiratory diseases in children (23 %). These are in line with common risk factors reported in three independent meta-analyses [28–30]. The first meta-analysis revealed a double risk of developing severe influenza complications (odds ratio 1.97, 95 % CI 1.06 – 3.67) when patients suffered from cardiovascular diseases compared to patients without [28]. In the second meta-analysis, cardiovascular diseases accounted for 17.2–20.0 % of all influenza-related hospitalizations [29], and in the third meta-analysis, influenza vaccination reduced the risk of severe complications by half (risk ratio 0.57, 95 % CI 0.39 – 0.82) [30]. The authors suggest that in patients with cardiovascular diseases, influenza may trigger the rupture of a vulnerable atherosclerotic plaque and thus lead to severe complications [30].

From societal perspective, the cost per episode is substantial. According to the current analysis, mean total costs in adults are about €514 per influenza episode and about €105 in children. Overall, the annual economic burden in Germany based on the present analysis of outpatient EMR data corresponds to the cost dimension published by Szucs *et al.* in 2001 [3].

The main cost driver in adults is indirect cost because of work days lost. Our findings are in accordance with the published literature [3, 5]. In adults with high cost episodes (> €1,000), more patients (90 %) were of working age compared to the total group (79 %) managed by PCPs. In the present analysis, the mean number of work days lost was 6.0 days in adults with recorded sick notes. This range is consistent with previous publications where 3.7–5.9 work days per episode have been reported lost following physician diagnosis of influenza [31].

The costs of seasonal influenza episodes were higher when patients were of working age and/or had experienced influenza-related complications. In the present analysis, influenza episodes in the working age group (17–59 years) were associated with mean total costs 2.4-times higher than those in patients ≥60 years. Complications almost tripled the cost per episode in children and increased the costs by a factor of 1.7 in adults. These findings are in line with results published by Karve *et al.* [4], where healthcare costs among influenza patients with complications were double those of influenza patients without complications.

In the present analysis, the majority of episodes with complications were reported in patients without risk factors. Adults without risk factors have traditionally not been included in influenza vaccination recommendations, which include only nursing-home residents, elderly individuals, and people at high risk for complications [1]. Consequently, a substantial burden occurs in patients outside the recommended group for receiving seasonal influenza vaccination. In Germany, healthy working adults have seasonal vaccination coverage of less than 16 % [32, 33].

If vaccination rates in healthy working adults were increased, the economic burden due to work days lost could be reduced substantially [34]. Based on a review of economic evaluations of seasonal influenza vaccination in healthy working age adults in the US, the authors conclude that vaccination does not generally save costs, but may be economically attractive under certain conditions, such as higher illness rates, lower costs of vaccination and higher wage rates [35]. To assess, whether vaccination is cost saving for a certain country, an evaluation based on country-specific epidemiological and health economic data is necessary.

In Germany, influenza vaccine recommendations for adults should be revisited in the future. The analysis of influenza surveillance by RKI during the 2012/2013 season showed that absence from work due to influenza reached the highest value for the last 10 years; there were more than 4 million reports of absenteeism [36]. Vaccination in children is widely discussed among clinicians [37] and a recent literature review concluded that vaccination in children, especially those below 5 years, reduces the influenza-associated disease burden [27]. Such reports emphasize the importance of targeting children and adults in national immunization policies.

There are limitations to the interpretation of our results. Overall, it has to be considered that the realization of the actual costs of seasonal influenza is challenged by the fact that the disease is underreported [38]. On the other hand, with respect to the cost per episode analysed in the present database analysis, the vast majority of episodes (over 92 %) was classified as “influenza, virus not identified” (ICD-10 J11). As the aim of the analysis was to describe the current economic burden, we did also consider the ICD-10 J11 diagnosis when describing the cost per influenza or ILI episode. Potentially, this introduces an underestimation of total costs, because ILI episodes might come with a milder course of the disease with shorter absence from work. Secondly, the total cost per episode is very likely to be underestimated because of missing information on emergency visits and admissions at hospitals in the *IMS* DA database, which is based on EMR data at office-based practices and not linked to hospital records. Also, information on patient expenses for OTC medication was not available in the EMR data. Thirdly, an underreporting of parental sick leaves is very probable. In children, sick certificates were recorded only for 3 episodes in the current analysis. According to a literature review in Western European countries, parental absenteeism is common (11–62 %) and lasts on average from 1.3 to 6.3 days in the parents of children with laboratory-confirmed influenza [26]. Additionally, co-payments were considered for all adult patients and without taking into account patient groups who do not have to co-pay, like those on chronic medication or unemployed. This results in a possible slight overestimation of co-payments. Finally, the extrapolation of cost per episode to annual economic burden of physician-attended influenza in Germany is a conservative calculation, because it was based on practice consultations and not on incidence figures. Therefore, an annual economic burden of up to €3.1 billion could be greatly underestimated.

## Conclusion

Findings demonstrate that in Germany, seasonal influenza is associated with a substantial clinical and economic burden. The current analysis provides an update of the economic burden associated with influenza and ILI in Germany based on a large patient sample derived from a representative primary care physicians’ panel. Nevertheless, the economic burden of influenza in Germany remains underestimated where influenza is underreported and comprehensive information on hospitalization and death related to influenza is not systematically recorded. Improving the effectiveness or coverage of influenza vaccination has the potential to reduce the burden of influenza.

## Additional file

Additional file 1:
**Unit cost and sources.**

## Abbreviations

ARE: Akute Respiratorische Erkrankungen [acute respiratory diseases]
CDC: Centers for Disease Control and Prevention
CI: Confidence interval
DM: Deutsche Mark
EBM: Einheitlicher Bewertungsmaßstab
ECDC: European Centre for Disease Prevention and Control
EMR: Electronic medical records
EU: European Union
ICD: International Classification of Diseases
ILI: Influenza-like illness
IMS DA: IMS Disease Analyzer
G-DRG: German diagnosis-related groups
OTC: Over-the-counter
PCP: Primary care physician
RKI: Robert Koch Institute
SD: Standard deviation
SHI: Statutory Health Insurance
STIKO: Staendige Impfkommission des Robert Koch Instituts [German Standing Committee on Vaccination]
US: United States
WHO: World Health Organization

## References

[1] World Health Organization (WHO). Influenza (Seasonal). Fact sheet N°211. March 2014 [http://www.who.int/mediacentre/factsheets/fs211/en/index.html, Access: 03.12.2014].

[2] Lai PL (2011). Burden of the 1999–2008 seasonal influenza epidemics in Italy - comparison with the H1N1v (A/California/07/09) pandemic. Hum Vaccin.

[3] Szucs T, Behrens M, Volmer T (2001). Volkswirtschaftliche Kosten der Influenza 1996. Eine Krankheitskostenstudie. Med Klin.

[4] Karve S, Misurski D, Herrera-Taracena G, Davis KL (2013). Annual all-cause healthcare costs among influenza patients with and without influenza-related complications: analysis of a United States managed care database. Appl Health Econ Health Policy.

[5] Levy E (1996). French economic evaluations of influenza and influenza vaccination. Pharmacoeconomics.

[6] Rychlik R, Heinen-Kammerer T, Rusche H, Piercy J, Scuffham P, Zollner Y (2003). Cost-effectiveness of prophylaxis and treatment of influenza. Dtsch Med Wochenschr.

[7] Scuffham PA, West PA (2002). Economic evaluation of strategies for the control and management of influenza in Europe. Vaccine.

[8] Jefferson T, Di Pietrantonj C, Rivetti A, Bawazeer GA, Al-Ansary LA, Ferroni E (2010). Vaccines for preventing influenza in healthy adults. Cochrane Database Syst Rev.

[9] European Center for Prevention and Disease Control (ECDC). Guidance Priority Risk Groups for Influenza Vaccination, Stockholm, August 2008 [http://ecdc.europa.eu/en/publications/Publications/0808_GUI_Priority_Risk_Groups_for_Influenza_Vaccination.pdf, Access: 28.08.2013]

[10] Grohskopf LA, Olsen SJ, Sokolow LZ, Bresee JS, Cox NJ, Broder KR, *et al.* “Prevention and Control of Seasonal Influenza with Vaccines: Recommendations of the Advisory Committee on Immunization Practices (ACIP) — United States, 2014–15 Influenza Season.” August 15, 2014 / 63(32);691–697. PMC4584910 25121712

[11] Robert Koch Institut. Epidemiologisches Bulletin 2013, Nr. 34 [http://www.rki.de/DE/Content/Infekt/EpidBull/Archiv/2013/Ausgaben/34_13.pdf?__blob=publicationFile, Access: 05.12.2013].

[12] Becher H, Kostev K, Schröder-Bernhardi D (2009). Validity and representativeness of the “Disease Analyzer” patient database for use in pharmacoepidemiological and pharmacoeconomic studies. Int J Clin Pharmacol Ther.

[13] Rothberg M, Haessler S, Brown R (2008). Complications of viral influenza. Am J Med.

[14] Haas W (2009). [Hrsg.]: Influenza.

[15] Einheitlicher Bewertungsmaßstab (EBM). 2012 [http://www.kbv.de/html/ebm.php]

[16] InEK GmbH – Institut für das Entgeltsystem 2012 (DRG Grouper) [http://www.g-drg.de]

[17] GKV Spitzenverband (National Association of Statutory Health Insurance Funds) 2012. http://www.gkv-spitzenverband.de/media/dokumente/krankenversicherung_1/krankenhaeuser/budgetverhandlungen/bundesbasisfallwert/BBFW_2012.pdf.

[18] Rote Liste 2012. Editio Cantor Verlag, Frankfurt/ Main 2012.

[19] Ehlken B, Ihorst G, Lippert B, Rohwedder A, Petersen G, Schumacher M, *et al*. Economic impact of community-acquired and nosocomial lower respiratory tract infections in young children in Germany. Eur J Pediatr. 2005;164:607–15. 10.1007/s00431-005-1705-0 15965766

[20] Statistisches Bundesamt 2012 [https://www.destatis.de/DE/ZahlenFakten/ZahlenFakten.html, Access: November 2012]

[21] von der Schulenburg JM G, Greiner W, Jost F, Klusen N, Kubin M, Leidl R (2008). German recommendations on health economic evaluation: third and updated version of the Hanover Consensus. Value Health.

[22] Robert Koch Institut. Berichte zur Epidemiologie der Influenza in Deutschland. [https://influenza.rki.de/Saisonbericht.aspx., Access: April 2014]

[23] an der Heiden M, Köpke K, Buda S, Buchholz U, Haas W. Estimates of excess medically attended acute respiratory infections in periods of seasonal and pandemic influenza in Germany from 2001/02 to 2010/11. PLoS ONE. 2013;8(7):e64593. Epub 2013/07/23. 10.1371/journal.pone.0064593 PMC3712969 23874380

[24] Robert Koch Institut. Saisonberichte 2010/11 [http://influenza.rki.de/Saisonberichte/2010.pdf, Access: 01.02.2013]

[25] Robert Koch Institut, Saisonberichte 2011/12 [http://www.rki.de/DE/Content/InfAZ/I/Influenza/PK_AGI_2012_AGI-Saisonbericht_11_12.pdf?__blob=publicationFile, Access: 01.02.2013]

[26] Antonova EN, Rycroft CE, Ambrose CS, Heikkinen T, Principi N (2012). Burden of paediatric influenza in Western Europe: a systematic review. BMC Public Health.

[27] Ruf BR, Knuf M (2013). The burden of seasonal and pandemic influenza in infants and children. Eur J Pediatr.

[28] Mertz D, Kim TH, Johnstone J, Lam PP, Science M, Kuster SP, Fadel SA, Tran D, Fernandez E, Bhatnagar N, Loeb M (2013). Populations at risk for severe or complicated influenza illness: systematic review and meta-analysis. BMJ.

[29] Mauskopf J, Klesse M, Lee S, Herrera-Taracena G (2013). The burden of influenza complications in different high-risk groups: a targeted literature review. J Med Econ.

[30] Udell JA, Zawi R, Bhatt DL, Keshtkar-Jahromi M, Gaughran F, Phrommintikul A, *et al*. Association between influenza vaccination and cardiovascular outcomes in high risk patients. A meta-analysis. JAMA. 2013;310:1711–20. 10.1001/jama.2013.279206 24150467

[31] Keech M, Beardsworth P (2008). The impact of influenza on working days lost: a review of the literature. Pharmacoeconomics.

[32] Blank PR, Schwenkglenks M, Szucs TD (2009). Vaccination coverage rates in eleven European countries during two consecutive influenza seasons. J Infect.

[33] Bohmer MM, Walter D, Muters S, Krause G, Wichmann O (2011). Seasonal influenza vaccine uptake in Germany 2007/2008 and 2008/2009: results from a national health update survey. Vaccine.

[34] Nichol KL (2001). Cost-benefit analysis of a strategy to vaccinate healthy working adults against influenza. Arch Intern Med.

[35] Gatwood J, Meltzer MI, Messonnier M, Ortega-Sanchez IR, Balkrishnan R, Prosser LA, *et al*. Seasonal influenza vaccination of healthy working-age adults: a review of economic evaluations. Drugs. 2012;72:35–48. 10.2165/11597310-000000000-00000 22191794

[36] Robert Koch Institut. Bericht zur Epidemiologie der Influenza in Deutschland Saison 2012/13 [https://influenza.rki.de/Saisonberichte/2012.pdf, Access: October 2013]

[37] Heikkinen T, Tsolia M, Finn A (2013). Vaccination of healthy children against seasonal influenza: a European perspective. Pediatr Infect Dis J.

[38] Molinari NAM (2007). The annual impact of seasonal influenza in the US. Measuring disease burden and costs. Vaccine.

