# Supplementary material for: Cost for physician-diagnosed influenza and influenza-like illnesses on primary care level in Germany – results of a database analysis from May 2010 to April 2012
Source: BMC Public Health. 2015 Jun 21;15:578. doi: 10.1186/s12889-015-1885-0 (PMC4475612; doi:10.1186/s12889-015-1885-0)
Supplement: Additional file 1: — Unit cost and sources. [file 12889_2015_1885_MOESM1_ESM.docx]

**Additional file: Unit cost and sources**

| **Cost component** | **Unit cost in €** | **Source** |
| --- | --- | --- |
|  |  |  |
| **Physician visit** |  |  |
| PCP |  |  |
| 17 to 59 years | 30.80 | EBM 2012 [15] |
| ≥ 60 years | 35.70 | EBM 2012 [15] |
| Pediatrician |  |  |
| ≤ 5 years | 41.65 | EBM 2012 [15] |
| 6 - 16 years | 30.80 | EBM 2012 [15] |
| **Drugs** | * | Rote Liste 2012 [18] |
| **Hospitalisation** |  |  |
| Base rate | 2992 | National Association of |
|  |  | Statutory Health Insurance Funds 2012 [17] |
| **Transportation** |  |  |
| Per physician visit | 6 | Ehlken 2005 [19] |
| **Loss of productivity per day** | 128.17 | Calculated by IMS |
|  |  | based on information from German Federal Statistical Office 2012 [20] |

* the respective price per prescribed drug according to the German official pharmaceutical index (Rote Liste) in 2012 was considered

PCP: Primary Care Physician; EBM: Einheitlicher Bewertungsmaßstab
